# Saxitoxin Poisoning in Green Turtles (*Chelonia mydas*) Linked to Scavenging on Mass Mortality of Caribbean Sharpnose Puffer Fish (*Canthigaster rostrata*-Tetraodontidae)

**DOI:** 10.3389/fvets.2019.00466

**Published:** 2019-12-17

**Authors:** Rocío González Barrientos, Gabriela Hernández-Mora, Fernando Alegre, Theresa Field, Leanne Flewelling, Sara McGrath, Jonathan Deeds, Yajaira Salazar Chacón, Karla Rojas Arrieta, Emilia Calvo Vargas, Karen Berrocal Artavia, Brian A. Stacy

**Affiliations:** ^1^Section of Anatomic Pathology, Department of Biomedical Sciences, College of Veterinary Medicine, Cornell University, Ithaca, NY, United States; ^2^Área de Bacteriología, Laboratorio Nacional de Servicios Veterinarios, Servicio Nacional de Salud Animal (SENASA), Ministerio de Agricultura y Ganadería, Heredia, Costa Rica; ^3^Jaguar Rescue Center, Playa Chiquita, Limón, Costa Rica; ^4^Ceiba Consulting Inc., Tampa, FL, United States; ^5^Florida Fish and Wildlife Conservation Commission, Fish and Wildlife Research Institute, St. Petersburg, FL, United States; ^6^US Food and Drug Administration, Center for Food Safety and Applied Nutrition, College Park, MD, United States; ^7^Unidad de Residuos y Contaminantes en Alimentos de Origen Acuático, Servicio Nacional de Salud Animal (SENASA), Laboratorio Nacional de Servicios Veterinarios, Departamento de lnocuidad de Alimentos, Ministerio de Agricultura y Ganadería, Heredia, Costa Rica; ^8^Laboratorio de Fitoplancton Marino, Escuela de Ciencias Biológicas, Estación de Biología Marina Juan Bertoglia Richards, Universidad Nacional, Puntarenas, Costa Rica; ^9^National Oceanic and Atmospheric Administration, National Marine Fisheries Service, Office of Protected Resources, University of Florida, Gainesville, FL, United States

**Keywords:** paralytic shellfish poisoning, sea turtle, biotoxin, fish kill, stranding, neurotoxin

## Abstract

Fish within the family Tetraodontidae are potential sources of both endogenous tetrodotoxins (TTXs) and dietary derived saxitoxins (STXs). Ingestion of fish tissues containing these toxins by other vertebrates can lead to severe illness and death. The Caribbean sharpnose puffer (*Canthigaster rostrata*) is a widespread tetraodontid species within the western Atlantic. Mass settlement of juveniles into foraging habitats have been associated with large-scale puffer fish mortality events. In 2013, 2014, and 2017, puffer mortality events on the southern Caribbean coast of Costa Rica were also associated with strandings of green turtles (*Chelonia mydas*) found to have fed on *C. rostrata*. Stranded sea turtles were found dead without apparent cause or alive with severe neurological signs that resolved during short periods of captivity. Puffer fish and turtle organ samples were analyzed for both TTXs and STXs. Concentrations of TTXs were extremely low in the fish (0.5–0.7 μg/g) and undetectable in turtle stomach contents. However, concentrations of STXs in whole fish (16.6–47.5 μg STX-eq/g) exceeded the 0.8 μg STX-eq/g human seafood safety threshold for STXs by orders of magnitude. Saxitoxins were also detected in samples of stomach contents (ingested fish), brain, lung, kidney, and serum from three affected turtles. Study results indicate that saxitoxicosis resulting from opportunistic foraging on *C. rostrata* during fish mortality events may be a significant factor in episodic stranding of green sea turtles in this region.

## Introduction

The Caribbean sharpnose puffer (*Canthigaster rostrata*: Tetraodontidae) is a common reef fish found in the western Atlantic. They are omnivorous and feed on a variety of benthic invertebrates (1). Large *C. rostrata* mortality events have been described in Costa Rica and other areas of the Caribbean (2, 3). Similar mass mortality is described in another *Canthigaster* species, Bennett's sharpnose puffer (*C. bennetti*) (4). These events involve juveniles of similar size and are hypothesized to result from stress, resource limitation, diseases, or other environmental factors during mass settlement events (2–4).

The tissues of tetraodontid puffers may contain tetrodotoxins (TTXs) (5) and also the chemically similar saxitoxins (STXs) (6, 7), which may result in morbidity or death if ingested in sufficient quantity. Tetrodotoxins are found naturally in some species of puffers and are hypothesized to be derived by certain marine bacteria, whereas STXs are produced in the marine environment by select dinoflagellates of the genus *Alexandrium* and *Pyrodinium* and bioaccumulate in higher trophic levels through the food chain. The occurrence, concentration, and relative tissue distribution of TTX and STX in puffers can vary greatly by species, even within the same system (8). Both of these toxins are a well-known human seafood safety concern in products intended for human consumption (9). To our knowledge, with the exception of laboratory studies in mammals, toxicosis from ingestion of tetraodontid puffers has only been observed in humans.

During 2013, 2014, and 2017, residents of the Limón Province on the southern Caribbean coast of Costa Rica observed mass mortality of *C. rostrata* coincident with strandings of green turtles (*Chelonia mydas*). Stranded turtles were found dead or alive with abnormal neurological signs. Here we present evidence that the turtles were exposed to and suffered ill effects of STXs, and not TTX, as the result scavenging on *C. rostrata* during fish mortality events.

## Materials and Methods

### Sea Turtle Evaluation and Sampling

Local residents of the Limón Province opportunistically record observations related to sea turtles and other marine life, including sea turtle strandings and other wildlife mortality. During the 2013 event, a recently deceased green sea turtle and fish samples were submitted to National Veterinary Service (SENASA) pathology laboratory for analysis. During the 2014 and 2017 events, live and deceased green turtles found during the events of this report were examined by a veterinarian from the Jaguar Rescue Center. Complete gross necropsies were performed both at SENASA and Jaguar Rescue Center on deceased turtles using standard techniques. Samples of gastric content and organs from dead turtles and puffers found on the beach were collected and frozen at −80°C until analysis.

### Biotoxin Analyses

We performed biotoxin analyses at different laboratories during the years of these events based on availability to receive samples and regional capability. A description of all samples, analyses, and laboratories is provided in Table 1. Puffers (*n* = 3) and samples collected from affected green turtles (*n* = 2) in 2013 and 2014 were imported into the U.S. (CITES permit nos. 13US724540/9, 2014-CR432/SJ, and 2014-CR433/SJ) for analyses at the Florida Fish and Wildlife Conservation Commission, Florida Fish and Wildlife Fish and Wildlife Research Institute (FWRI) and US Food and Drug Administration, Center for Food Safety and Applied Nutrition (CFSAN). A pooled sample of approximately 10 juvenile puffers and stomach contents (partially digested puffers) from a deceased green turtle collected during a subsequent mortality event in April-May 2017 were analyzed for STXs in Costa Rica by the Unit of Residues and Contaminants in Food of Aquatic Origin of the Department of Food Safety of the National Laboratory of Veterinary Services (LANASEVE), National Service of Animal Health, Ministry of Agriculture and Livestock. In total, samples analyzed for STX included gastric contents (partially digested pufferfish) from single turtles collected during events in 2013, 2014, and 2017, serum and tissues from the turtle necropsied from the 2013 event, and analysis of puffers collected from the environment during the 2013 and 2017 events. Analysis for TTX was only conducted on puffers collected during the 2013 event.

**Table 1:** Inventory of samples analyzed for saxitoxins (STXs) and tetrodotoxin (TTX) by mortality event, laboratory, and method.

**Year**	**No. of turtle strandings**	**Sampled**	**Sample type**	**Laboratory**	**Method(s)**
2013	52	Turtle (*n* = 1)	Stomach content, serum, lung, kidney, brain	FWRI, CFSAN	STX ELISA, STX-HPLC-FL^a^, LC-MS/MS (STX and TTX)^a^
		Puffers (*n* = 3)	Whole—individual	FWRI, CFSAN	LC-MS/MS (STX and TTX)
2014	Unknown	Turtle (*n* = 1)	Stomach content	FWRI	STX ELISA, STX-HPLC-FL
2017	37	Turtle (*n* = 1)	Stomach content	LANASEVE	STX-HPLC-FL
		Puffers (*n* = 10)	Whole—pooled	LANASEVE	STX-HPLC-FL

*Laboratories included the Florida Fish and Wildlife Conservation Commission, Florida Fish and Wildlife Fish, and Wildlife Research Institute (FWRI), US Food and Drug Administration, Center for Food Safety and Applied Nutrition (CFSAN), and Unit of Residues and Contaminants in Food of Aquatic Origin of the Department of Food Safety of the National Laboratory of Veterinary Services (LANASEVE), National Service of Animal Health, Ministry of Agriculture and Livestock. Methods included enzyme-linked immunosorbent assay (ELISA) and high-performance liquid chromatography with fluorescence detection (HPLC-FL) for detection of STXs and high-performance liquid chromatography-tandem mass spectrometry (LC-MS/MS) for detection of STXs and TTX. Stomach contents consisted of ingested puffers*.

a *Stomach contents only*.

For STX analysis at FWRI, tissues were homogenized, and 5 ml of 0.1 M HCl was added to a 5 g aliquot of each sample. The mixture was adjusted to pH 2.5–4, boiled for 5 min in a water bath, and centrifuged at 3,000 × g for 10 min. The supernatant was retained, and a 0.5 g equivalent was passed through a pre-conditioned SPE C18 cartridge (3 ml, 500 mg). The effluent was collected, the cartridge was rinsed with 2 ml deionized water, and the rinse water was combined with the effluent. Extracts were assayed for STXs using the Abraxis Saxitoxin (PSP) ELISA (enzyme-linked immunosorbent assay). Samples analyzed at LANASEVE were extracted using 0.1 M HCl as described above and were deproteinated using trichloroacetic acid (30% v/v) as described in Rourke et al. (10). At both labs, samples were analyzed using high performance liquid chromatography with fluorescence detection (HPLC-FL) and post-column oxidation (10, 11). Saxitoxin congeners were identified and quantified by comparison to certified reference solutions of saxitoxin (STX), neosaxitoxin (neoSTX), gonyautoxin-1/4 (GTX-1/4), GTX-2/3, 21-N-sulfocarbamoyl saxitoxin (GTX-5/B1), decarbamoyl saxitoxin (dcSTX), dcGTX-2/3, and C1/2 purchased from National Research Council Canada.

Analyses of puffer fish from the 2013 mortality event for both TTX and STX was conducted at CFSAN using high-performance liquid chromatography-tandem mass spectrometry (LC-MS/MS). Three fish ranging in size from 0.8 to 1.5 g were analyzed independently for toxin content by extracting twice with 10 mL each of 1% acetic acid/water through homogenization with a Polytron model PT 10–35 immersion dispenser fitted with a model PTA 20/2W generator (Kinematica, Switzerland). Combined supernatants were brought up to 20 mL with 1% acetic acid/water. Samples were then filtered by syringe fitted with a 0.22 mm filter and diluted 1:10 with 1% acetic acid/water before analyzing by LC-MS/MS. Contents from one turtle stomach collected in 2013 (30 g total) were analyzed for toxin content by homogenizing as described above followed by extraction as described in Deeds et al. (8). Due to the more complex nature of the sample, 2 g of homogenized stomach contents (containing mainly partially digested puffers) were extracted twice with 10 ml each of 1% acetic acid/methanol followed by centrifugation at 3,000 × g. Combined supernatants were taken to dryness under a stream of N_2_ and reconstituted in 10 mL of 1% acetic acid/water. Two mL of the final suspension were defatted with 7 ml of chloroform by vortexing followed by centrifugation at 2,500 × g. The aqueous extract was then filtered and diluted 1:10 with 1% acetic acid/water as described above. Extracts were analyzed by LC-MS/MS using a Shimadzu Nexera X2 LC30 UPLC coupled to an 8,050 triple quadrupole mass spectrometer. The UPLC was equipped with a TSK-gel amide 80 column (2.1 × 15 cm) and operated in isocratic mode, with a mobile phase of 65% acetonitrile/35% water/50 mM formic acid/2 mM ammonium formate flowing at 0.4 mL/min. Based on initial HPLC-FL findings, the mass spectrometer was configured to monitor 3 transitions each of toxins TTX, STX, dcSTX, and GTX-5, with a single transition for each used for quantitation (m/z: 320.1 –> 161.95 for TTX, 300.1 –> 282.1 for STX, 257.1 –> 126.1 for dcSTX, 380.1 –> 300.15 for GTX-5).

### Phytoplankton Analyses

Water samples were collected from the area of the puffer mortality in October 2013 (09.63847, −82.69389) and analyzed at the Laboratorio de Fitoplancton Marino, Escuela de Ciencias Biológicas, Universidad Nacional. Surface water samples were collected at 1 and 5 m and preserved with Lugol's solution. The quantitative analysis of microalgae was carried out using the Utermöhl method with 10 mL columns and an inverted microscope. The concentration of the microalgae were expressed in number of cells/L. To our knowledge, no water samples were collected during the other events.

## Results

### Field Observations and Sampling

**Figure 1:**
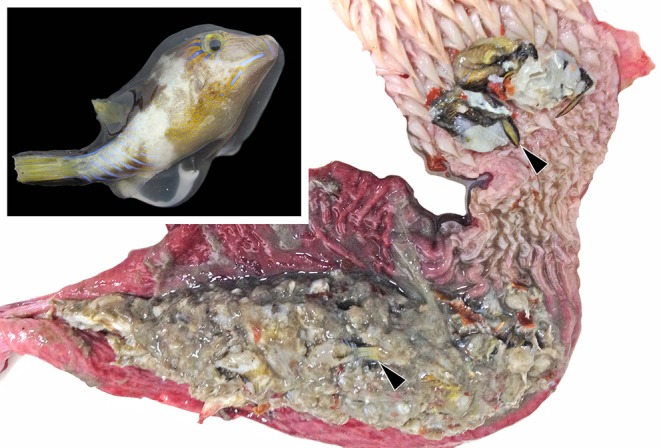
The esophagus and stomach of a green turtle (*Chelonia mydas*) found dead during mass mortality of Atlantic sharpnose puffer fish (*Canthigaster rostrata*, inset) on the Caribbean coast of Costa Rica in November 2013. The lumen is opened showing the remains of multiple puffers, the tails of which are recognizable (arrowheads).

In September 2013, large-scale mortality involving thousands of *C. rostrata* was reported in Limón Provence, first in Punta Uva and then involving beaches in Cocles and Gandoca. Concurrent with these reports were observations of stranded green turtles and dogs and cats becoming ill after feeding on dead puffers. Based on information obtained from a local Sistema Nacional de Áreas de Conservación (SINAC) official, we estimate that approximately 52 green turtles were found stranded during the period of puffer mortality. Most were found dead and in various states of decomposition. Two live green turtles exhibiting paralysis or extreme paresis were taken into captivity and placed into shallow water to prevent drowning. Both animals recovered within 24–48 h to the degree that they could be released. Additional diagnostic evaluation and treatment was not pursued due to limited resources in the area. One juvenile male green turtle (43 cm from nuchal notch to tip of the suprapygal) in good postmortem was found floating and was collected for necropsy. This animal was in good nutritional condition and had abundant froth throughout the respiratory tract (interpreted as seawater aspiration). Multiple *C. rostrata* were found within the caudal esophagus and partially digested remains of the same species filled the stomach (Figure 1). Histopathological findings were regarded as incidental to the cause of death and included moderate numbers of spirorchiid trematode ova embolized within multiple tissues with mild associated histiocytic inflammation, as well as gastric and enteric lymphoplasmacytic inflammation attributable to endoparasitism.

Three additional pufferfish mortality events concurrent with green turtle strandings were reported in the same region in 2014 and 2017. Thirty-seven green sea turtles were found dead during April and May 2017 between Playa Negra de Puerto Viejo to Gandoca. Two of these animals, one very decomposed and one recently deceased, were necropsied (gross examination only) and found to have many partially digested *C. rostrata* within the esophagus and stomach. Samples of partially digested puffers from the non-decomposed turtle and a few *C. rostrata* found in the area were collected for biotoxin analysis. The other two events occurred during May 2014 and November 2017 but reliable data on numbers of turtles affected are not available. A single decomposed green turtle was collected during the 2014 event and upon necropsy *C. rostrata* were found in its stomach and were sampled for analysis. No turtle carcasses or pufferfish were collected for analysis during the event in November 2017.

### Biotoxin Analysis

Results of analyses for STXs and TTX for samples collected during all events are shown in Tables 2 and 3. Saxitoxins were detected in all of the turtle organs and stomach contents analyzed by ELISA, and were confirmed in turtle stomach contents and in all puffer fish analyzed using HPLC-FL or LC-MS/MS, depending on the year (Table 1). For both HPLC-FL and LC-MS/MS analyses of samples collected in 2013 and 2014, the saxitoxin toxin profile observed was dominated by STX (57–99%), with GTX-5 present in all samples (trace-40%), and low levels in dcSTX (trace-9%) found in all but one sample (Tables 2, 3). Tetrodotoxin concentrations were extremely low in whole puffer fish, and TTX was not detected in the puffers retrieved from the stomach contents of the single green turtle collected in 2013 as determined by LC-MS/MS. In 2017 samples analyzed by LANASEVE, saxitoxin (51–57%), GTX-5 (6–9%), and dcSTX (4–6%) were also observed as well as C1 (21–25%) and C2 (9–12%) (Table 2).

**Table 2:** Results of high-performance liquid chromatography with fluorescence detection (HPLC-FL) analyses of samples from stranded green sea turtles (*Chelonia mydas*) collected in 2013, 2014, and 2017 and puffer fish (*Canthigaster rostrata*) from 2017.

**Specimen**	**Sample**	**ELISA STX eq**	**HPLC-FL total STX eq^a^**	**Toxin molar %**
Turtle (2013)	Stomach content	0.69	0.53	STX 64%; GTX-5 36%; trace dcSTX
	Serum	0.05	–	–
	Lung	0.05	–	–
	Kidney	0.04	–	–
	Brain	0.04	–	–
Turtle (2014)	Stomach content	0.40	0.44	STX >99%; GTX-5 trace; dcSTX 0%
Turtle (2017)	Stomach content	–	1.93	STX 51%; C1 25%; C2 12%; GTX-5 6%; dcSTX 4%
Pooled puffer fish (2017)		–	7.86	STX 57%; C1 21%; C2 9%; GTX-5 7%; dcSTX 6%

*Individual organs from turtles were first analyzed for saxitoxins (STXs) by enzyme-linked immunosorbent assay (ELISA) (reported as saxitoxin equivalents, 2013 and 2014 only). Values shown are micrograms of saxitoxins per gram of homogenized sample or per ml of serum. Dashes indicate those samples that were not analyzed using a given method*.

a *Converted using the toxicity equivalency factors of Oshima (12) of STX: 1, dcSTX: 0.51, GTX-5: 0.06, C1: 0.006, C2: 0.096*.

**Table 3:** Results of high-performance liquid chromatography-tandem mass spectrometry (LC-MS/MS) analyses of Caribbean sharpnose puffer fish (*Canthigaster rostrata*) collected during a mortality in 2013 that was concurrent with strandings of green sea turtles (*Chelonia mydas*).

**Specimen**	**LC-MS/MS Total STX eq^a^**	**Toxin molar %**	**TTX**
Puffer 1 (2013)	16.64	STX 72%; GTX-5 24%; dcSTX 4%	0.07
Puffer 2 (2013)	22.79	STX 67%; GTX-5 30%; dcSTX 3%	0.05
Puffer 3 (2013)	47.45	STX 57%; GTX-5 40%; dcSTX 3%	0.07
Ingested puffers (2013)	0.78	STX 62%; GTX-5 31%; dcSTX 7%	ND

*Whole dead fish collected from the beach and ingested fish removed from the stomach of a deceased turtle were analyzed for paralytic shellfish toxins (saxitoxin [STX], decarbamoyl saxitoxin [dcSTX], and GTX-5) and tetrodotoxin (TTX). Values shown are micrograms of toxin per gram of homogenized sample (ND-not detected)*.

a *Converted using the toxicity equivalency factors of Oshima (12) of STX: 1, dcSTX: 0.51, GTX-5: 0.06*.

### Phytoplankton Analyses

No algal blooms or water discoloration events were reported during the mortality events. Low concentrations (100 cells per L) of two species of dinoflagellate, *Prorocentrum micans* and *Protoperidium divergens*, were present in water samples collected near the end of the 2013 event.

## Discussion

Although our sample sizes were very small due to logistical constraints within the study area, we were able to identify a probable cause of the sea turtle strandings and link them with episodic mass mortality of *C. rostrata*. Our evidence includes demonstration of foraging on puffers by green turtles, detection of relatively high concentrations of STXs in the dead puffers, toxin absorption by turtles, documentation of neurological abnormalities in live stranded turtles, and exclusion of other apparent causes of sea turtle stranding based on a limited number of examined animals. Affected turtles ultimately may have died from neurological dysfunction or drowning secondary to toxicosis as evidenced in our necropsy findings. Saxitoxicosis in green turtles during these events appears to have resulted from opportunistic foraging on puffers that died or were moribund. The affected life phase of green turtle is primarily herbivorous; however, they will opportunistically consume easily available sources of fish in some circumstances and readily take fish in captivity (Stacy pers. obs).

The concentration of STXs measured in *C. rostrata* ranged from 16.64 to 47.45 μg STX-eq/g. These concentrations exceed the human seafood safety threshold (0.8 μg/g) by orders of magnitude (9) and are within the range of concentrations reported in other saxitoxin contaminated tetraodontid puffers responsible for human poisoning events (6–8). Lethal doses for STXs have not been defined for sea turtles or other reptiles, but comparative data from other taxa provide some context for our observations. Oral LD50 values for STXs in mammals and birds range from 91 to 263 μg/kg (13). Based on the average concentration of STXs detected by LC-MS/MS in homogenized fish tissues [29.0 μg STX-eq/g, calculated using experimentally derived toxicity factors for each congener as reported in (12)] and the average body weight of individual dead fish [0.9 g, (3)], ingestion of 3–9 fish per kg would provide enough toxin to reach the LD50 range determined for other taxa. We estimate that number of fish ingested by turtles easily exceeded this dose as the stomachs of necropsied turtles contained the remains of dozens of *C. rostrata* in addition to an unknown number that likely were digested beyond recognition. Toxin concentrations were lower in gastric contents than whole fish, which could reflect degradation or absorption.

Sea turtle mortality associated with STX-producing dinoflagellate blooms is reported from the Pacific coast of Mexico and Central America, and Papua New Guinea (14–17). These published accounts reported higher STX toxin concentrations in individual turtle tissues (1.16–4.78 μg STX eq/g) than STXs we detected in green turtles feeding on *C. rostrata* (approximately 0.04 μg STXs/g). Mortality due to suspected saxitoxicosis also has been reported in an estuarine turtle, the diamondback terrapin (*Malaclemys terrapin*), with lower concentration of STXs (0.002–0.12 μg STX eq/g) than we detected in green turtle tissue and gastric contents (18). The differences in toxin concentrations among reports could be attributable to methodology, metabolism, as well as dose, timing, and duration of exposure. Data are too limited at this time to inform interpretation of toxin values alone with regard to toxicological effect; rather they serve to demonstrate toxin exposure that should be interpreted in the context of clinical signs, postmortem findings, and environmental data.

Due to their genetic resistance to TTX and STX (19), tetraodontid puffer fish are potential reservoirs of both STXs and TTXs (8). The biological origin of STX in puffer fish has not been defined, but may result from ingestion of toxin-containing prey species or STX-producing dinoflagellates (7). Studies have measured these toxins in puffers caught for research or consumption; there are no reported ill effects on the fish themselves.

To our knowledge, no STX-producing blooms were reported during the period and area of our observations and water samples associated with the 2013 event did not contain STX-producing dinoflagellates; however, sampling was very limited, and we were unable to find any other available data on phytoplankton monitoring for these areas. Documented STX producers in the Caribbean Sea include *Pyrodinium bahamense, Gymnodinium catenatum*, and *Alexandrium tamarense* (20, 21). Notably, the limited STX profile observed in 2013 and 2014 samples (STX, dcSTX, and GTX-5) correspond to a similar report of STX accumulation in southern puffers (*Sphoeroides nephalus*) in Florida, USA responsible for human poisonings (7). This report linked STXs in southern puffers to blooms of the dinoflagellate *Pyrodinium bahamense* based on co-occurrence and the similar toxin composition in both algae and puffer tissues. The profiles reported in 2017 samples also included C1 and C2, which may indicate a different PSP species played a role in this event. The Landsberg et al. (7) study showed that STX containing *S. nephalus* retained toxicity in captivity for an extended period, suggesting that the STX observed in *C. rostrata* reported here could have originated from an undetected dinoflagellate bloom that occurred prior to the mortality events.

The cause of episodic or periodic mass mortality of *C. rostrata* has not been specifically defined but may be related to mass recruitment of juveniles into foraging areas, as has been reported in other tetraodontid puffers (3, 4). This mortality may be part of species natural history; however, reports of mortality during mass settlement of various fishes cite other considerations in need of further study, including diseases and environmental factors (3, 22).

Our report describes a unique pathway of saxitoxin exposure, linking mass mortality of a common reef fish and biotoxin reservoir with morbidity and death of scavenging sea turtles. Additionally, saxitoxin poisoning has not been previously documented in sea turtles in the Caribbean. Further studies of the drivers of *C. rostrata* mass mortality and opportunistic scavenging by green turtles, as well as the sources of the STXs themselves, are needed in order to better understand the occurrence of these events.

## Data Availability Statement

All datasets generated for this study are included in the article/supplementary material.

## Ethics Statement

The animal study was reviewed and approved by University of Florida Institutional Care and Use Committee.

## Author Contributions

All authors contributed to data and sample collection and review and preparation of the manuscript. Sample and data analyses were conducted by BS, LF, RB, GH-M, SM, JD, YC, KR, KA, and EV.

### Conflict of Interest

TF works for Ceiba Consulting, Inc. The remaining authors declare that the research was conducted in the absence of any commercial or financial relationships that could be construed as a potential conflict of interest.
